# Impact of School Nurse on Managing Pediatric Type 1 Diabetes with Technological Devices Support: A Systematic Review

**DOI:** 10.3390/diseases12080173

**Published:** 2024-08-01

**Authors:** Giovanni Cangelosi, Stefano Mancin, Sara Morales Palomares, Paola Pantanetti, Elisabetta Quinzi, Giulia Debernardi, Fabio Petrelli

**Affiliations:** 1 Units of Diabetology, ASUR Marche, 63900 Fermo, Italy; dr.paolapantanetti@gmail.com; 2 Humanitas Research Hospital, 20089 Rozzano, Italy; stefano.mancin@humanitas.it; 3 Department of Pharmacy, Health and Nutritional Sciences (DFSSN), University of Calabria, 87036 Rende, Italy; sara.morales@unical.it; 4 Nursing Home Fermo Seminary, 63900 Fermo, Italy; elisabetta.quinzi@gmail.com; 5 AUSL Bologna, Ospedale Maggiore “C.A. Pizzardi”, 40133 Bologna, Italy; giuliadebernardi93@gmail.com; 6 School of Medicinal and Health Products Sciences, University of Camerino, 62032 Camerino, Italy; fabio.petrelli@unicam.it

**Keywords:** school nurse, pediatric population, educators, type 1 diabetes, technological devices, public health

## Abstract

Introduction: Type 1 diabetes (T1D) is a disease that primarily occurs in pediatric populations. A school nurse (SN) can provide valuable support in the school setting for minors affected by this condition. Methods: The main objective of this study was to evaluate the impact of nursing care provided to adolescents and children with T1D using technological devices in school. Qualitative and quantitative outcomes considered in the included studies were collected and discussed. A systematic review was conducted in the PubMed, CINAHL, and Scopus databases and reported thought the PRISMA guidelines. Results: Eleven studies were included. The results showed that SNs need to enhance both their skills and organization to effectively manage young patients with T1D using technology. The response of both the pediatric population and their caregivers to the disease management by a SN has been positive. Conclusions: The management of chronic diseases is one of the most urgent public health issues, especially for Western healthcare systems. Proper management of patients with T1D at the school level is definitely an aspect that policymakers and healthcare managers should consider to improve the quality of life of this extremely vulnerable population, particularly those using technological management T1D.

## 1. Introduction

The World Health Organization (WHO) defines diabetes as one of the worst killers of our time [1,2]. Type 1 diabetes (T1D) is an autoimmune disease where the immune system mistakenly destroys insulin-producing cells in the pancreas, causing a severe lack of insulin needed to control blood sugar levels. Genetics often contribute to T1D, and environmental factors can also trigger it. Despite research, the exact causes of T1D are still unclear. T1D usually starts in children and adolescents but can occur at any age, including adulthood [3,4,5]. COVID-19 infection, along with various environmental factors, appears to have significantly contributed to an exponential increase in this condition. Recent studies suggest that SARS-CoV-2 may trigger the autoimmune destruction of β-cells, potentially increasing the incidence of T1D [6,7]. In addition, questionable behaviors such as smoking, alcohol consumption, sedentary lifestyle, and improper diet play a decisive role in the management and glycemic compensation of both T1D and other forms of diabetes, including Type 2 diabetes (T2D) and gestational diabetes [8,9,10,11,12,13,14,15]. There is increasing evidence supporting the clinical management of T1D through technology [16,17,18]. About 1.52 million people worldwide are affected by T1D. In Italy, according to data from the Italian National Institute of Statistics (ISTAT) in 2018, there are approximately 300,000 individuals with T1D. Of these, about 10% use Continuous Subcutaneous Insulin Infusion (CSII), and only 39% use devices for Continuous Glycemic Control (CGM) [5,6,19,20]. The other 51% of individuals with T1D usually manage their condition using multi-injected insulin therapy. A more modern approach to managing T1D in all aspects of daily life, especially for populations like the younger generation, is increasingly recommended and supported by solid evidence in favor of technological devices [21,22,23,24,25,26]. In this context, a school nurse (SN) could act as a true manager of care in heterogeneous contexts, both in terms of resources and management [27,28,29]. For these main reasons, specific pathways have been developed in recent years, especially nursing-oriented ones, for clients using CSII and CGM who increasingly require highly specialized care [30]. This review aims to explore the role of SNs in managing pediatric patients with T1D who use technological devices in a school setting.

## 2. Materials and Methods

### 2.1. Review Methodology

This systematic review followed a pre-registered protocol on the Open Science Framework (https://doi.org/10.17605/OSF.IO/Z9GF8, last accessed on 24 June 2024) and was reported according to the PRISMA (Preferred Reporting Items for Systematic Reviews and Meta-Analyses) guidelines [31] to improve rigor and transparency in mapping the existing literature.

### 2.2. Objectives

The main objective of the study was to evaluate the care provided by SNs to young individuals with T1D using technological devices in an educational setting. Secondly, the collected quantitative and qualitative results were analyzed. The review aimed to address the following research questions:

- How does the SN interact in a school context with individuals with T1D using technology?
- How does the SN support care and relationships among all parties involved in the complex assistance in the clinical setting under consideration?

### 2.3. Formulation of the Research Question

The research questions for this review were formulated using the PICOS (Population, Intervention, Comparison, Outcome, Study design) framework [32], specifically P, Pediatric population 0–18 years old (children/adolescents/young people) with T1D using devices and/or SNs and/or parents/caregivers involved in the care pathway; I, nursing care in the school setting for pediatric populations with T1D using technological devices; C, interventions vs. different interventions and/or vs. no intervention; O, qualitative and quantitative outcomes; and S, primary literature studies.

### 2.4. Search Strategy

Searches were performed in the PubMed/Medline, Scopus, and CINAHL databases. Three academic researchers (G.C., G.D., and E.Q.) independently participated in the study selection process, and a fourth academic researchers (S.M.) was consulted for final selection and dispute resolution. 

For this study, search strings were created using keywords from database thesauruses such as MeSH, as well as additional free terms. The keywords included “school nurse”, “child”, “adolescents”, “young”, “educators”, “T1D”, “CGM”, “Insulin Devices”, “artificial pancreas”, “Insulin Pump (IP)”, “CSII”, and “Diabetes Technological Device”, along with their variations, combined strategically using Boolean operators AND and OR (Supplementary File). 

### 2.5. Criteria and Process

The inclusion criteria encompassed primary literature studies involving a pediatric population (0–18 years) with T1D using devices and/or sensor networks and including parents or caregivers in the care pathway. The studies had to investigate nursing care in the school setting utilizing technological devices, with comparisons made between different interventions or between interventions and no intervention, reporting both qualitative and quantitative outcomes.

The exclusion criteria ruled out studies involving adults ≥18 years, lacking device or SN use, or not involving parents or caregivers. Studies not set in schools, not involving technological devices, without comparison groups, not reporting relevant outcomes, or comprising secondary literature were excluded.

### 2.6. Evaluation of Risk of Bias and Methodological Quality of Studies

The risk of bias and methodological quality of the included records were independently assessed by two researchers using the Joanna Briggs Institute (JBI) critical appraisal tools, adhering to the JBI framework methodology [33]. Any disagreements were resolved by an impartial third researcher.

### 2.7. Data Extraction and Synthesis

Data from the selected records were extracted and reported in a specific data extraction table including authors, year of publication, country, study design, populations, outcome analysis, and results. The study results were reported as a narrative summary, summarized according to the review objectives and integrated with figures and tables.

## 3. Results

**Figure 1:**
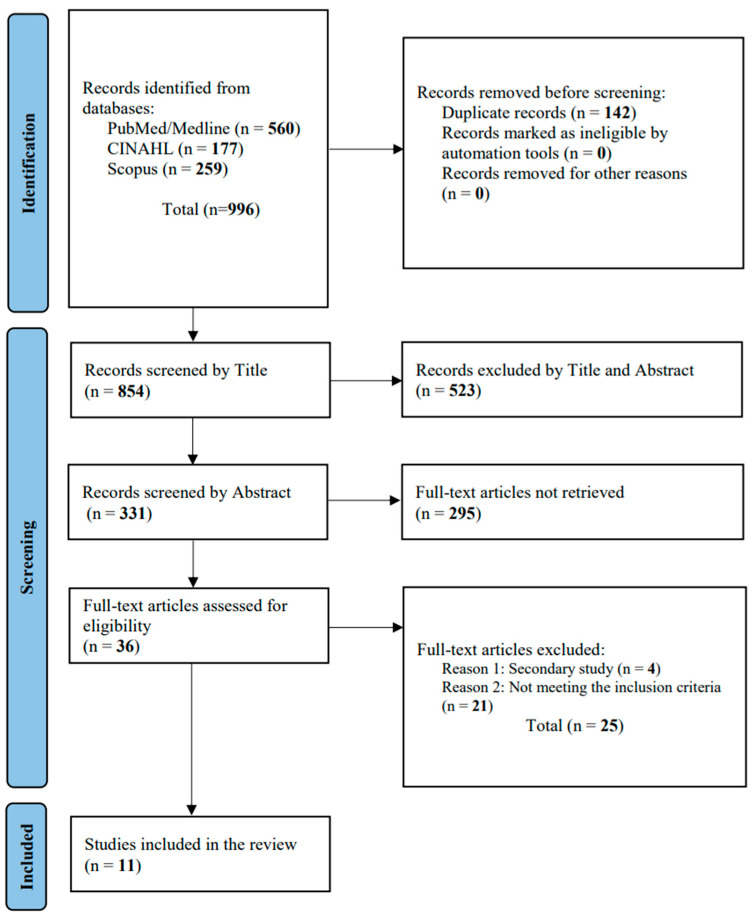
PRISMA flowchart.

A comprehensive search of the databases found 996 articles: 560 from PubMed—MEDLINE, 177 from CINAHL, and 259 from Scopus. After removing 142 duplicates, 854 titles were screened. Of these, 331 abstracts were evaluated, and 295 were deemed irrelevant, leaving 36 full-text articles. After further assessment, 25 were excluded for not meeting our criteria. Finally, 11 studies were included in this systematic review (Figure 1).

### 3.1. Characteristics of the Studies Included

The included studies were conducted as follows: six in the United States of America (USA), two in Australia, one in Italy, one in Saudi Arabia, and one in Poland. Overall, they involved 1579 subjects: 157 students, 645 parents/caregivers, and 777 SNs/educators. Three studies were conducted with student populations (one including parents’ analyses), three with parents/caregivers, and five with SNs/educators. Only one study was conducted using randomized controlled trial (RCT) methodology (Table 1). All included studies demonstrated adequate methodological quality and low risk of bias.

### 3.2. Students

In a feasibility study by Tonyushkina KN et al. [34], the ability to adjust monthly insulin doses in youths using IP was evaluated through collaboration between the reference pediatric diabetes clinic and SN. Thirty students from a low-income community, ranging from elementary to high school age, were enrolled. The students were supported in device management by a specialized SN. After two years, only 27% of them regularly downloaded device data. The absence of SN support appears to be a negative factor in the treatment process. Glycemic trends showed improvements during the school year compared to the summer period, with a decrease in glycated hemoglobin (HbA1c) values from 8.4% to 11.0%. Engelke MK et al. [35] conducted a descriptive study exploring the care provided to children with T1D by a SN, highlighting differences based on nurse workload and child age. They recruited 86 students (aged 5–17) from various ethnic backgrounds attending both public and private school in the USA. SNs assigned to one to two schools provided more intervention days (mean 40.3) compared to those responsible for three to four institutes (mean 24.4, *p* < 0.05). The study found that 25 students faced school emergencies that were handled by parents or teachers in the absence of a SN. According to the study, the presence of a SN seems strategic, especially in managing emergencies, promoting overall health, and enhancing the well-being of teens with T1D with device support. In a RCT conducted by Izquierdo R et al. [36], telemedicine management and support for T1D were compared to conventional care in 41 school age children (5–14 years): 18 in the control group and 23 in the experimental group. In the telemedicine approach, in addition to the usual care, specific technological equipment was provided through which the healthcare professional could collaborate synchronously with the SN from the reference diabetes center and exchange blood glucose information digitally. This approach resulted in a decrease in urgent calls, and 91% of the children expressed high levels of satisfaction with this service, indicating their willingness to use it again based on a questionnaire. Only 9% expressed dissatisfaction with SN services. The HbA1c values increased in the first 6 months for students in the usual care group and decreased in the intervention cohort (*p* < 0.02).

### 3.3. Parents/Caregivers

To assess whether children with T1D in Saudi Arabia are adequately supported in their care within the school setting, Alaqueel AA [37] conducted a cross-sectional study using an online platform, distributing surveys to parents of children aged 4 to 19 years old with T1D. A total of 411 parents responded to the questionnaire; only 36 of the children used IP (8.8%). The survey investigated who administered insulin at school, whether meals were adequate, the availability of glucagon, and peer interactions towards children with T1D. The findings indicated that only 8.6% of school staff were qualified to provide T1D care, and SNs were a predominantly marginal figure, leading to children often self-administering insulin. Moreover, 8% of children did not receive insulin during school hours, likely because 79% did not have a specific treatment plan at school. Even though 90.3% of children regularly visited an endocrinologist, 89.4% still had uncontrolled HbA1c levels. These findings suggest that the current standard of care for children with T1D is inadequate, highlighting the need for better community-based care. Pinelli L et al. [38] conducted a qualitative study using a semi-structured questionnaire to assess how Italian parents manage children with T1D (6 and 13 range years) during school time. The study investigated aspects such as insulin administration, hypoglycemia management, and the use of glucagon. Two hundred and twenty individuals completed the survey, revealing that only in 3.6% of cases there was a SN in the school care setting, while in 2.9% of cases, a teacher took responsibility for managing insulin therapy. In the remaining percentage of cases, children self-administered insulin or received assistance from a parent; 55.9% of respondents did not believe that their child’s school was adequately equipped to handle T1D emergencies. Marks et al. [39] conducted a qualitative research project through semi-structured telephone interviews with 14 Australian mothers of children in their first or second year of primary school (6–7 years old) with T1D, 10 of whom were using IP. The interviews explored insulin administration during school hours, the role of parents, and their confidence in the school’s ability to manage T1D. The results indicate that the Australian education system lacks adequate healthcare support structures for children with T1D and is practically devoid of the figure of the SN, placing the entire management responsibility on parents. The survey participants expressed concerns about their children’s safety in the school environment and emphasized the need for qualified SNs who could provide assistance for T1D, especially for those using technological devices.

### 3.4. Nurses/Educators

In the study by Pinelli L et al. [38], in addition to Italian parents of children with T1D, qualitative research and a semi-structured survey were conducted with 52 teachers. The study found that 40.4% of teachers received specific training on T1D, but only 2.9% were willing to take responsibility for treating the condition, and 23% of the sample believed that their schools were adequately equipped to handle emergencies. The study demonstrates that educators are not adequately trained to assist children with T1D (especially those using technology), although there were no reported incidents of negligence or mismanagement during emergencies. Therefore, training sessions should be provided for school staff who are willing to be supported by a SN for T1D treatment.

March et al. [40] developed a new unidimensional scale initially comprised of 50 questions, which was later reduced to 25, in order to assess the knowledge of SNs regarding devices for the treatment of T1D, such as IP. The scale was administered to 310 SNs from Pennsylvania. The purpose was to analyze proficiency in usage, identify training gaps, and consequently customize interventions for SNs. The results showed that 95% of the sample had experience with IP and 92% with CGM, but only 34% had worked with integrated care systems. In another study, March et al. [41] conducted a cross-sectional survey consisting of 12 items directed at 132 nurses. Only 104 professionals, including 78 SNs and 26 district nurses, provided complete and sufficient responses for analysis. The study found that only 23% of SNs had specific experience with integrated technological care systems for children with T1D, suggesting the need for school-specific guidelines for using these advanced products, which would benefit children and teens. In addition, March et al. [42] conducted semi-structured interviews with 40 SNs from American public elementary and secondary schools. These interviews showed the high potential of modern diabetes management devices but also significant gaps in training. SNs expressed a need for more specific education and greater collaboration with school staff and teachers.

**Figure 2:**
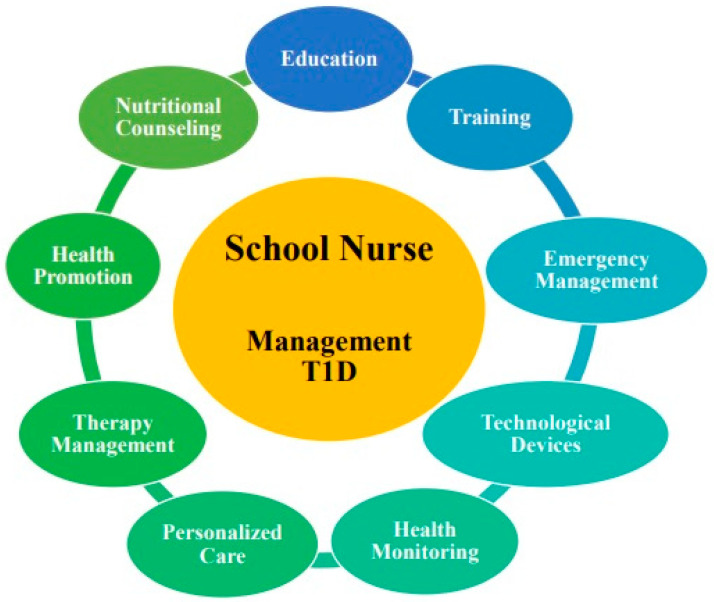
Main competencies of the SN in managing T1D. SN = school nurse; T1D = type 1 diabetes.

Kobos et al. [43] conducted a cross-sectional study to assess the actual and perceived knowledge among SNs from 17 primary schools in Poland. The questionnaire was administered to 230 SNs focusing on their general knowledge of diabetes, insulin and glucagon, IP, diabetes complications, nutrition, physical activity, stress, comorbidities, and blood glucose measurements. Correct responses averaged 46.7%, with the lowest scores in the knowledge of technological devices for T1D care (36.5%), nutrients (37.4%), and insulin and glucagon (37.9%). The actual and perceived knowledge about diabetes were moderately correlated. SNs who scored the highest were graduates, those with relatives and friends with T1D, and with previous specific topic training. Finally, a qualitative and narrative inquiry was conducted on 13 diabetes educators (DEs) caring for children with T1D attending the first 3 years of an Australian primary school [44]. Telephone interviews, conducted by a researcher and SN, discussed the use of IP in children aged 4 to 8, focusing on improving support during school hours. The interviews primarily revealed barriers regarding the use of IP in the school system, the need for supportive adults during school hours, and the importance of specific training for this condition with technological devices for both SNs and DEs. Figure 2 provides a graphical representation of the main results.

## 4. Discussion

Chronic diseases have become one of the most significant concerns for healthcare policymakers in recent years, primarily due to their primary and secondary complications [45,46,47,48,49,50]. Given the nearly uncontrollable global and national growth trends, diabetes (both type 1 and type 2) requires increasing attention to specific aspects, such as diet and exercise, as it is transforming into a global epidemic [51,52,53,54,55,56,57,58,59,60]. Integrated care strategies that consider the patient as a whole, rather than focusing solely on the “diabetological” aspect, should be the most common proposals for managing the disease at all levels of care [61,62,63,64,65,66]. A modern, multidimensional/multidisciplinary approach is now the essential organizational tool on which public health officials should rely for an overall improvement in qualitative and quantitative outcomes [67,68,69,70,71,72,73,74].

Our research underscores the necessity for enhanced coordination in managing chronic diseases, especially for patients requiring specialized therapeutic education. This is particularly true for children with T1D using technological devices in school environments. Nurses play a crucial and strategic role in achieving better technical and emotional compliance with integrated care systems. A specific study observed that CSII management, supported by SNs, led to improved glycemic control during the school year compared to the summer [34]. This highlights the critical role of consistent SN involvement in optimizing diabetes management. Previous studies have similarly demonstrated that SN interventions are linked to better adherence to treatment protocols and improved health outcomes [75]. Additionally, research conducted in countries like Saudi Arabia, Italy, and Australia indicates a prevalent lack of sufficient support for children with T1D, often due to the limited or nonexistent presence of SNs [37,38,39]. This often forces children to self-administer insulin, resulting in suboptimal HbA1c levels and overall inadequate diabetes management during school hours. In many instances, the primary responsibility for managing T1D falls on the parents or the children themselves, highlighting structural and resource deficiencies in schools to effectively handle diabetes-related emergencies.

These challenges extend beyond T1D and are also evident in the management of other common chronic conditions among children, such as asthma. International studies have demonstrated that sufficient healthcare support, particularly from trained SNs, can greatly enhance asthma management [76]. Educating school staff to identify and respond to asthma emergencies is essential for preventing severe medical incidents and ensuring student safety. Similar to T1D, effective asthma management necessitates collaboration between schools, parents, and healthcare professionals to develop personalized treatment plans and provide continuous support [77].

Ultimately, the nurse’s role is essential and strategic in enhancing both the technical and emotional adherence to integrated care. Schools and their staff should actively support children, yielding substantial social and community benefits. Type 1 diabetes (T1D) impacts patients’ lives both clinically and emotionally, regardless of age. Establishing appropriate clinical pathways within the right care settings can significantly bolster the psychological health of vulnerable individuals, particularly at the onset of the disease.

Indeed, there is a global shortfall in personal care within sensitive areas like schools and communities, a problem worsened by the management difficulties experienced during the COVID-19 pandemic [78,79,80,81,82,83,84]. This situation calls for the scientific community to consider enhancing support structures for chronic disease patients. Nurses, particularly family and community nurses, should be adequately valued and held accountable, as they could effectively bridge organizational gaps in primary care [85,86,87,88]. Therefore, it would be worthwhile to explore the potential for family and community nurses to develop their skills further for the community’s benefit. Enhanced the training and empowerment of these professionals could significantly improve chronic disease management, fostering a safe and inclusive educational environment for all students. Integrating specific skills into the roles of existing nurses could provide a practical and immediate solution to these challenges, ultimately improving the quality of life for children with T1D and other chronic conditions [89].

### Limitations

The study reveals several limitations. In the included studies, the aspect of technological devices and the presence of SNs were not always clearly and consistently structured, and only in some were they the main focus of the researchers. Standardizing interventions and study populations was also challenging due to the extreme heterogeneity in healthcare systems, timelines, outcomes considered, and research designs. Therefore, it was not possible to combine the outputs and conclude with a summary meta-analysis. Furthermore, we encountered challenges regarding the consistent reporting of insulin administration methods across the included studies. This variability prevented us from providing a clear and detailed breakdown in Table 1. To maintain the integrity of our data synthesis and mitigate potential reporting biases, we opted to not address these data. Similarly, while blood glucose and HbA1c measurements were integral to our analysis, their comprehensive inclusion across all 11 studies was hindered by inconsistencies in reporting. Therefore, our synthesis of the findings reflects the available data extracted from narrative discussions within the reviewed literature. Nonetheless, the obtained results certainly require careful interpretation but still allow for reaching clear conclusions that could stimulate further scientific research on the matter.

## 5. Conclusions

This systematic literature review highlights the significant role of the SN, a nurse specialty already established in some Western countries. In regions where SNs are present, both students and educators benefit from more efficient management, though there are areas requiring reassessment, particularly in the management of T1D using technological devices. Healthcare organization is often lacking, compounded by deficiencies in school systems where professionals frequently lack specific training, especially for emergency situations. In nearly all cases, parents remain the primary caregivers for students, revealing critical gaps in modern, integrated care systems. Effective student integration necessitates addressing both the health and social aspects, urging health managers to develop specific care pathways for students of all ages and socioeconomic backgrounds living with T1D and using technological support. In this context, the SN can act as a central figure within a multidisciplinary and multidimensional approach to daily clinical practice. By establishing and empowering the role of SNs, it is possible to bridge existing gaps and provide comprehensive and effective care. This integration of the educational environment with essential health support enhances the overall well-being of students with T1D.

## Figures and Tables

**Table 1:** Synthesis studies included.

Author (Year)	Population Study/Country	Cohort	Type of Study and Times	Outcome Analysis	Results
Tonyushkina KN et al. (2021) [34]	Students/USA	30 students from elementary to high school from low-income communities	Pilot single arm study	feasibility of monthly insulin adjustments, HbA1c levels and Quality of life	After two years, 27% of the subjects downloaded the device data. The absence of the SN appears to be a negative factor for treatments.
Engelke MK et al. (2011) [35]	Students/USA	86 students (5–17 age)	Descriptive study	HbA1c levels and Quality of life	For the teens, case management by SN can improve quality of life.
Izquierdo R et al. (2009) [36]	Students/USA	41 students (5–14 age)	RCT	HbA1c levels and Quality of life	A telemedicine group (23), supported by SNs showed improvements over the first 6 months compared to those receiving usual care (18). 91% of the students would reuse telemedicine services.
Alaqueel AA et al. (2019) [37]	Parents/Saudi Arabia	411 parents of child and teens (3–19 years)	Cross sectional study	HbAlc and Carbohydrate Counting	Only 8.8% of children use an IP, and 8.6% of school staff are trained, according to the sample’s opinion, in treating T1D, and 79% of children do not have a written plan at school for managing T1D.
Pinelli L et al. (2011) [38]	Parents and teachers/Italy	220 parents and 52 teachers of child (6–13 years)	Qualitative study	Management of T1D in school setting	The 40.4% of teachers received specific training on the condition, but only 2.9% would be willing to take responsibility for treating it. SNs are present in the care setting in only 3.6% of cases.
Marks AL et al. (2020) [39]	Parents/Australia	14 mothers of child (6–7 years)	Qualitative Study	Management and knowledge of T1D in school setting with technology device	The Australian education system lacks adequate healthcare support structures for children with T1D who use technological devices. The presence of SNs is virtually absent in the setting analysis.
March CA et al. (2022) [40]	SN/USA	310 SN	Qualitative Study	Management and knowledge of T1D in school setting with technology device	The 95% of SNs had experience with IP, 92% with CGM, but only 34% had worked with integrated disease management systems.
March AC et al. (2021) [41]	SN/USA	132 SN	Cross sectional study	Management and knowledge of T1D in school setting with technology device	The 23% of SNs reported having experience with integrated IP at school, while 46% had no knowledge of them. The 82% of cohort study emphasized that children and teens should be supported in using these products at school.
March CA et al. (2020) [42]	SN/USA	40 SN from primary and secondary level schools	Qualitative Study	Management and knowledge of T1D in school setting with technology device	The study highlighted educational areas as the main focus for potential improvement in school support for students with T1D using technological devices.
Kobos E et al. (2020) [43]	SN/Poland	230 SN who have worked in schools in the past year	Cross-sectional study	Management and knowledge of T1D in school setting with technology device	The rate of correct responses was 46.7%, 36.5% were familiar with CSII, 37.4% with proper nutrition, and 37.9% with glucagon and insulin.
Marks A et al. (2018) [44]	Diabetes Educators (DEs)/ Australia	13 Educators of child in primary school (4–8 years)	Qualitative study	Management of T1D in school setting	Despite numerous barriers, Australian DEs implemented varied methods to facilitate the use of technology for T1D therapy.

Legend. SN = school nurse, HbA1c = glycated hemoglobin, RCT = randomized controlled trial, IP = insulin pump, T1D = type 1 diabetes, CGM = continuous glucose monitor, CSII = continuous subcutaneous insulin infusion, DEs = diabetes educators.

## Data Availability

Data supporting this research are available in the manuscript and Supplementary File.

## Supplementary Materials

The following supporting information can be downloaded at https://www.mdpi.com/article/10.3390/diseases12080173/s1: Supplementary File (search strategy).
