# Supplementary material for: Impact of School Nurse on Managing Pediatric Type 1 Diabetes with Technological Devices Support: A Systematic Review
Source: Diseases. 2024 Aug 1;12(8):173. doi: 10.3390/diseases12080173 (PMC11353902; doi:10.3390/diseases12080173)
Supplement: Supplementary file 1 [file diseases-12-00173-s001.zip › diseases-3072567-supplementary.pdf]

Supplementary File

(Search strategy update June 26, 2024)

PubMed Strategy

((((((((school nurse) OR (school nurses)) OR (school nurse role)) OR (school nursing)) OR (school nursing services)) OR (public school nursing)) OR (nursing school education)) OR (nursing school curriculum)) OR ((((("School Nursing"[Mesh]) OR ("Nurses, Community Health"[Mesh] OR "Nurse Specialists"[Mesh])) OR "Nurses, Public Health"[Mesh]) OR ("Public Health Nursing"[Mesh] OR "Paediatric Nursing"[Mesh])) OR "Education, Nursing"[Mesh])) AND ((((((((((((((((((glucose monitoring) OR (CGM)) OR (insulin device)) OR (insulin devices)) OR ("Insulin Infusion Systems"[Mesh])) OR ("Pancreas, Artificial"[Mesh])) OR ("Biomedical Technology"[Mesh] OR "Technology Assessment, Biomedical"[Mesh] OR "Medical Informatics"[Mesh])) OR (artificial pancreas)) OR (artificial pancreas type 1)) OR (artificial pancreas system)) OR (artificial pancreas diabetes)) OR (insulin pump)) OR (insulin pumps)) OR (insulin pump therapy)) OR (continuous glucose monitoring)) OR (diabetes cgm)) OR (type 1 diabetes cgm)) OR (CGM)) OR (cgm diabetes)))) Filters: Clinical Trial

Total results: 560

Scopus Strategy

((TITLE-ABS-KEY (insulin AND pump) OR TITLE-ABS-KEY (glucose AND monitoring) OR TITLE-ABS-KEY (glucose AND control) OR TITLE-ABS-KEY (glucose AND monitoring AND device) OR TITLE-ABS-KEY (glucose AND device) OR TITLE-ABS-KEY (cgm) OR TITLE-ABS-KEY (artificial AND pancreas) OR TITLE-ABS-KEY (insulin AND pump AND therapy) OR TITLE-ABS-KEY (cgm AND education) OR TITLE ABS KEY (cgm AND child AND education) OR TITLE-ABS-KEY (cgm AND adolescent AND education) OR TITLE-ABS-KEY (insulin AND pump AND device))) AND ((TITLE-ABS-KEY (school AND nurse) OR TITLE-ABS-KEY (school AND nursing) OR TITLE-ABS-KEY (school NAD nursing AND intervention) OR TITLE-ABS-KEY (school AND nursing AND practice) OR TITLE-ABS-KEY (school AND nurse AND role) OR TITLE-ABS-KEY (public AND nurse AND school) OR TITLE-ABS-KEY (public AND health AND nursing AND promotion) OR TITLE-ABS-KEY (public AND health AND nursing)))

Total results: 259

Cinhal strategy

1 AND 2

1. (school nurse or school health nurse or school nursing) OR (school nurse or school health nurse or school nursing) OR school nursing OR school nurse intervention OR school nurse role OR school

nurses challenges OR (school nursing and health promotion) OR school nursing evidence-based clinical practice guideline: medication administration in schools OR school nursing practice OR public health nursing OR (public health nursing or community health nursing) OR public health nurse

2. glucose monitoring OR (glucose monitoring or glucose control or glycaemic control or sugar control) OR glucose monitoring device OR artificial pancreas OR artificial pancreas is 'life-changing' for children with diabetes OR cgm OR (cgm in adolescents and young adults with type 1 diabetes) OR insulin pump therapy OR cgm education OR cgm use in adolescents OR insulin pump OR (insulin pumps and school and paediatrics)

Total results: 177
